# GABA_A_ receptor encephalitis associated with human parvovirus B19 virus infection: Case report

**DOI:** 10.1097/MD.0000000000026324

**Published:** 2021-06-11

**Authors:** Daniel Almeida do Valle, Mara Lúcia S. Ferreira Santos, Mônica J. Spinosa, Bruno A. Telles, Carolina Prando, Mara L. Cordeiro

**Affiliations:** a Faculdades Pequeno Principe; b Neurology Department, Hospital Pequeno Principe; c Instituto de Pesquisa Pelé Pequeno Principe; d Radiology Department, Hospital Pequeno Principe; e Immunology Department, Hospital Pequeno Príncipe, Curitiba, Brazil; f Department of Psychiatry and Biobehavioral Sciences, University of California Los Angeles, CA.

**Keywords:** encephalitis, γ-aminobutyric acid type A receptor antagonists, human parvovirus B19

## Abstract

**Rationale:**

Human parvovirus B19 (B19) infection can produce a spectrum of clinical syndromes, including neurological manifestations, most notably encephalitis. Although symptoms suggestive of autoimmune disease in patients with B19 infection have been previously described, a clear association of autoimmune encephalitis with B19 infection has yet to be established.

**Patient concerns:**

We describe the case of a 6-year-old boy who was hospitalized due to status epilepticus, which evolved to super-refractory status epilepticus that was only mildly responsive to anticonvulsant drugs.

**Diagnosis:**

A cerebrospinal fluid study identified slight pleocytosis and B19 positivity. A subsequent autoimmunity cerebrospinal fluid study revealed the presence of anti-γ-aminobutyric acid type A (GABA_A_) receptor antibodies.

**Interventions:**

After pulse therapy with methylprednisolone and continuous therapy with prednisolone with cyclosporine, the patient experiencing seizure persistence with disordered motor function manifestations and only minor improvement in consciousness, and so, plasmapheresis was performed. With continued immunosuppressive treatments with cyclosporine and prednisolone, the patient's clinical picture showed progressive improvement, with good control of seizures. Although the patient tolerated withdrawal of the anticonvulsant drugs well, he developed seizures when corticosteroid therapy withdrawal was attempted, so was started on azathioprine.

**Outcomes:**

After immunosuppressive therapy, the patient evolved with complete remission of symptoms, normal neurological examination and age-appropriate neuropsychomotor development.

**Lessons:**

The present case characteristics, together with previous findings, support the hypothesis that autoimmunity may be triggered by extensive antigen release due to degeneration of infected neurons. This case highlights the importance of early clinical suspicion and treatment.

## 1 Introduction

Human parvovirus B19 (B19) has been linked to a broad spectrum of clinical syndromes, including syndromes with neurological manifestations, especially encephalitis.^[1]^ Classically, a rash has been considered a typical characteristic of B19 infection, though it is not always present. Indeed, there are no definite distinguishing features of B19-associated encephalitis compared with other forms of viral encephalitis.^[1]^

Patients with B19 infection often exhibit signs suggestive of an autoimmune pathology, such as chorea, cerebellar ataxia, and status epilepticus (SE). Moreover, there are anecdotal reports of these symptoms being treated effectively with human immunoglobulin (Ig) and/or steroid therapies. However, there is a lack of scientific literature regarding a possible association of autoimmune encephalitis with this virus.^[2]^

Here we describe the case of a previously healthy child with a confirmed diagnosis of B19 encephalitis who was found to have antibodies targeting γ-aminobutyric acid type A (GABA_A_) receptors.

## 2 Case presentation

A previously healthy 6-year-old boy was evaluated in our emergency department. Initially, he presented with a history of headache for the past 10 days (including the evaluation day), after experiencing a self-limited and focal impaired-awareness seizure. Two days later, the patient was brought back and admitted to the hospital for focal seizures characterized by left-sided facial twitching without impaired awareness. Physical and neurological examination without abnormal findings. The seizure was treated immediately with benzodiazepine midazolam (0.2 mg/kg/dose), and he was prescribed phenobarbital (10 mg/kg/d) and phenytoin (15 mg/kg/d). He was submitted to a cranial computerized tomographic scan, the results of which showed no abnormalities.

Because the patient continued to experience seizures after being on the above medications for 2 days, the treatment plan was switched to carbamazepine (30 mg/kg/d) and topiramate (15 mg/kg/d). After 5 days of this new treatment, the seizures evolved to persistent seizures, requiring oro-tracheal intubation and intensive care unit (ICU) admission. After 2 days in the ICU, the patient exhibited focal SE together with fluctuating consciousness (Glasgow coma scale score range, 3–12). A pathology hypothesis of encephalitis and acute disseminated encephalomyelitis was made. Accordingly, treatment with intravenous antiviral pharmacotherapy (acyclovir, 30 mg/kg/d) and the steroid methylprednisolone (30 mg/kg/d for 5 days) was additionally prescribed.

**Figure 1:**
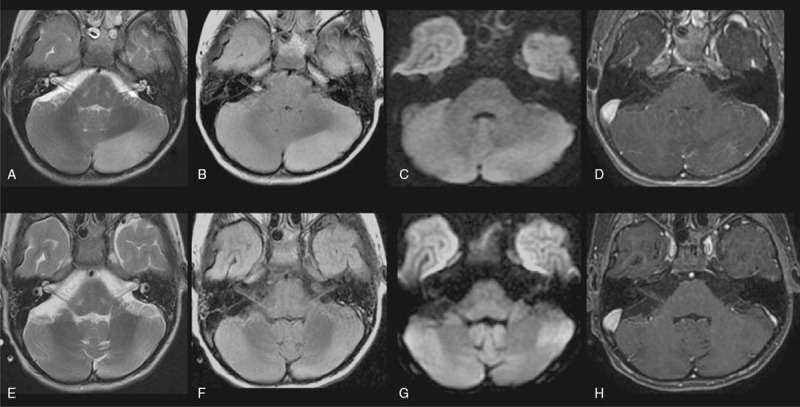
Brain MRI of a pediatric patient with autoimmune anti-GABA_A_ receptor encephalitis comorbid with an established B19 infection of the central nervous system at two timepoints. A–D. Images from the patient's first MRI scan, which occurred while he was being treated in the ICU for persistent seizures that evolved into focal SE with fluctuating consciousness. T2/FLAIR hyperintensity and mild expansion in the left cerebellar hemisphere, with some foci of contrast enhancement and an absence of restricted diffusion. E–H. Images from a follow-up scan performed almost a month later, likely showing residual post-inflammatory changes. Note that the previously observed T2/FLAIR hyperintensity and volume expansion were reduced and that there were no new lesions or areas of contrast uptake in the left cerebellar hemisphere.

Brain magnetic resonance imaging (MRI) (Fig. 1A–D) performed on his 15th day in the ICU demonstrated T2/FLAIR hyperintensity and a mild expansion of the left cerebellar hemisphere, with some contrast enhanced foci and an absence of restricted diffusion. These findings were strongly suggestive of a pronounced inflammatory and/or infectious process, such as acute cerebellitis. A cerebrospinal fluid (CSF) study revealed slight pleocytosis (14 cells/mm^3^), CSF positivity for B19 (polymerase chain reaction test), and immunonegativity for tumor cell markers. Rheumatologic and immunological tests with ferritin augmentation yielded normal results.

Continuous midazolam (2 μg/kg/min) suppressed the onset of clinical seizures, though self-limited electrographic seizures continued to occur. Upon discontinuation of continuous midazolam, clinical seizures restarted, characterized by right lip and right upper limb movements without loss of consciousness even in the presence of various antiepileptic drugs (phenytoin, phenobarbital, valproic acid, topiramate, and carbamazepine). After pulse therapy with methylprednisolone (30 mg/kg/d for 5 days), prednisolone was maintained at 2 mg/kg/day together with cyclosporine at 4 mg/kg/d. A video electroencephalogram performed during a seizure revealed a non-epileptogenic nature of the facial and upper limb myoclonic events. The patient developed hypertensive peaks that could be controlled with amlodipine (0.6 mg/kg/d) and enalapril (0.6 mg/kg/d).

Despite the above treatments, the patient developed mental confusion and extremity tremors. Five sessions of plasmapheresis were performed on alternate days, yielding substantial progressive improvement of tremors, complete recovery of consciousness, and recovery of sitting and ambulation. Although the child regained consciousness, he maintained speech impairment and had difficulty naming his parents as well as objects, numbers, and letters. Additionally, he exhibited left hemibody hyperreflexia with cerebellar ataxia that impaired his ability to walk unassisted.

A follow-up brain MRI scan performed a month after the first MRI scan showed reductions in T2/FLAIR hyperintensity and volume loss, relative to the prior scan, without new lesions or contrast uptake in the left cerebellar hemisphere (Fig. 1E–H). These findings were suggestive of residual post-inflammatory changes. A concomitant CSF study revealed the presence of autoantibodies targeting the γ-aminobutyric acid type A (GABA_A_) receptor.

With continued immunosuppressive treatments with cyclosporine (4 mg/kg/d) and prednisolone (2 mg/kg/d), the patient's clinical picture showed progressive improvement, with good control of seizures and systemic hypertension. Although the patient tolerated withdrawal of the anticonvulsant drugs well, he developed seizures when corticosteroid therapy withdrawal was attempted. To control seizures upon corticosteroid therapy withdrawal, the patient was started on azathioprine (1.7 mg/kg/d), which stabilized his condition, enabling him to be discharged from the hospital.

Three months later, the patient was hospitalized for severe respiratory distress and fever. Treatment with piperacillin-tazobactam was initiated together with staggered antibiotic therapy, but the patient failed to stabilize. He was diagnosed with interstitial pneumonia, which resolved in response to a 12-day course of meropenem, vancomycin, micafungin, and sulfamethoxazole-trimethoprim, allowing him to be discharged.

**Figure 2:**
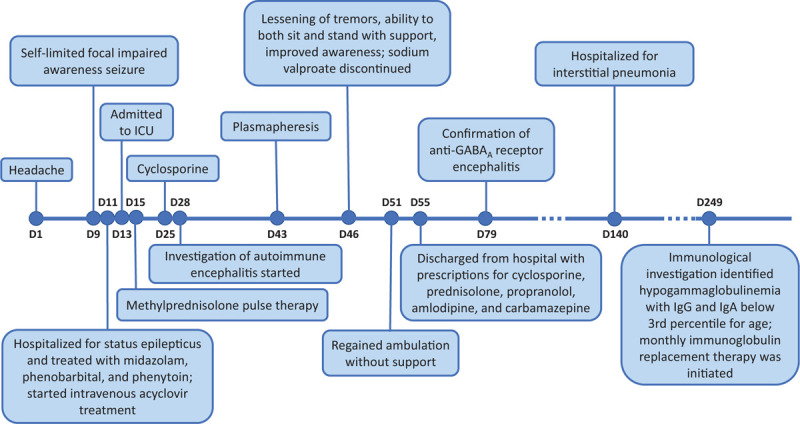
Timeline with treatment progression and diagnosis time. D, day; ICU = intensive care unit; IgG, IgA, immunoglobulin.

One month later, the patient was readmitted to start immunosuppressant therapy with cyclophosphamide. While hospitalized, he presented with febrile neutropenia without a microbial or clinical focus. Immunological investigation identified hypogammaglobulinemia, characterized by IgG (326 mg/kg) and IgA (<10 mg/kg) levels below the 3rd percentile for his age with a normal IgM level (55 mg/kg). Whole exome sequencing did not reveal any pathogenic mutations in genes responsible for primary immunodeficiency. Ig replacement therapy was initiated; the patient was put on monthly Ig infusions in addition to maintenance immunosuppression with the aforementioned daily azathioprine treatment. Figure 2 summarizes the major signs and symptoms as well as the pharmacological management.

At the time of writing of this report, the patient (now 9 years and 10 months old) had been maintained for 22 months on monthly Ig infusion and azathioprine, with no new infections or symptom recurrences. Although he has continued to show persistent leukopenia during this follow-up period, he has maintained normal neutrocyte counts. Importantly, the patient has normal neurological examination results indicating age-appropriate neuropsychomotor development. The study was approved by the Institutional Ethics Committee on human research at the Little Prince Children's Hospital, Curitiba, Brazil (# CAAE:41965220.5.0000.0097). Written informed consent was obtained from the patient's parents to report the case and to use the MRI scans for publication.

## 3 Discussion

Here, we report a peculiar case of a pediatric patient with medically recalcitrant focal epilepticus disease that was secondary to anti-GABA_A_ receptor encephalitis. The patient's disease was associated with CSF polymerase chain reaction positivity for B19 and MRI findings suggestive of acute cerebellitis.

Autoimmune encephalitis with antibodies against synaptic GABA_A_ receptors is a severe form of encephalitis characterized by seizures that are prone to evolving into refractory SE.^[3]^ Typically, GABA_A_ receptor antibody-associated encephalitis presents with refractory epilepsy or continuous partial epilepsy.^[4,5]^ Other symptoms of the disease that have been described in the literature include cognitive impairment, psychiatric symptoms, and disordered movement.^[4,6]^ In a study of 26 patients with anti-GABA_A_ receptor autoimmunity, identified from a population of 2,914 patients with suspected autoimmune neurologic disorders, Spatola et al.^[7]^ found that all 26 patients had seizures accompanied by at least one of the following core symptoms: cognitive impairment, decreased level of consciousness, altered behavior, or movement disorder. Our patient's profile of presenting with seizures together with an altered level of consciousness and subsequent development of a movement disorder characterized by cervical dystonia was consistent with Spatola et al's observations.^[7]^

Differential diagnosis of autoimmune encephalitis due to anti-GABA_A_ receptor immune activity is made by anti-GABA_A_ receptor antibody detection in serum and CSF. In some cases, antibodies may be detected exclusively in serum.^[7]^ Ionotropic GABA_A_ receptors mediate most of the fast-inhibitory synaptic transmission in the brain and play a critical role in regulating brain excitability. The GABA_A_ receptor is a ligand-gated ion channel formed by combinations of different subunit subtypes: α (1–6), β (1–3), γ (1–3), δ, ε, θ, π, and ρ (1–3).^[7,8]^ The main epitope targets of anti-GABA_A_ receptor antibodies have been reported to be the α1and β3.^[7]^ Physiologically, anti-GABA_A_ receptor antibodies may change the distribution of GABA_A_ receptor subtypes or reduce GABA_A_ receptor quantities.^[9]^

The etiology of anti-GABA_A_ receptor encephalitis is variable, sometimes being associated with tumors, predominantly in adults, and sometimes being associated with infections, mainly in the pediatric population.^[4]^ Viral infection of the central nervous system may facilitate the development of an autoimmune response targeting neurotransmitter receptors. Interestingly, in the aforementioned study by Spatola and colleagues,^[4]^ 2 patients had developed GABA_A_ receptor autoimmunity encephalitis within weeks of developing herpes simplex encephalitis (one infected with herpes simplex virus type 1 and one infected with herpes simplex virus type 6). Remarkably, these 2 patients exhibited *de novo* antibody synthesis against both N-methyl-D-aspartate receptors and GABA_A_ receptors, which would not be expected to occur if the autoimmunity had been consequent to a viral mimicry mechanism.^[4]^

In the present case, our patient was confirmed to have a CSF infection with B19. The precise pathogenesis underlying the development of B19 encephalitis and encephalopathy is unclear. Proposed etiological hypotheses for this phenomenon include direct viral toxicity, accumulation of toxic, virally-encoded NS1 protein, cytokine dysregulation, and autoantibody production directed against brain antigens.^[2,10]^ The production of antibodies against self-antigens and the induction of inflammatory cytokine production in the presence of B19 infection has been suggested to be consequent to molecular similarities between host and viral proteins.^[11,12]^ Previously, patients with parvovirus infections have been found to develop anti-N-methyl-D-aspartate receptor encephalitis.^[13,14]^ The findings in these cases and the current case are consistent with the hypothesis that autoimmune reactions may be triggered in B19 infected patients by the release of large quantities of neuronal antigens produced by neuronal degeneration secondary due to Parvoviridae viral infection of the central nervous system.

With respect to imaging, brain MRI results for patients with GABA_A_ receptor autoimmunity encephalitis may sometimes be normal; otherwise, patients often exhibit multifocal, non-diffusion-restricting, non-enhancing medium-to-large sized cortical, juxtacortical, and subcortical lesions, usually in the temporal lobe.^[4,5]^ These MRI changes may be consequent to immune activity or prolonged seizures.^[7]^ There is no clear correlation of the presence of neuroimaging alterations with clinical severity or prognosis, with some asymptomatic patients presenting with neuroimaging alterations and some patients with brain lesions exhibiting improvement with treatment.^[4,6]^ The patient in the present case presented with a single cerebellar lesion that had normalized about a month after ongoing anticonvulsant pharmacotherapy was supplemented with steroid treatments. The limited scope and subsequent normalization of neuroimaging changes observed in this case may reflect his relatively early diagnosis and treatment.

A particularly challenging aspect of managing the care of our patient was his persistent seizures and eventual SE, the treatment of which involved multiple anticonvulsant drugs and induced coma in the ICU. Seizures that are secondary to autoimmune encephalitis are often refractory to antiepileptic drugs unless the underlying immune mechanism is identified and treated. In many cases, as in the present one, SE is an early manifestation of the disease.^[7]^ Our patient's symptoms improved markedly after plasmapheresis, corroborating the notion that autoimmune encephalitis should be treated with immunosuppressive therapy. Notwithstanding, this patient suffered from seizure recurrences upon withdrawal of the therapy underscoring the fact that the appropriate duration for immunosuppressive therapy in patients with autoimmune encephalitis is unknown.^[15]^

Because Ig therapy was introduced after our patient had already been treated with immunosuppressive drugs in the present case, we could not establish whether his subsequently detected hypogammaglobulinemia was a primary or secondary condition. Indeed, at the time of the examination that revealed hypogammaglobulinemia, the patient was taking multiple medications that can induce hypogammaglobulinemia, including prednisolone, azathioprine, and carbamazepine. The fact that whole exome sequencing did not reveal any abnormalities in the present case suggests that it is likely that the hypogammaglobulinemia was secondary to immunodeficiency. Ig replacement therapy is indicated in cases of recurrent infection episodes and concomitant low IgG levels.^[16,17]^

## 4 Conclusion

The present case findings, together with previous findings, support the hypothesis that antigens released due to the degeneration of virally infected neurons can trigger the pathogenesis of an autoimmune disease. The present case also highlights the importance of early clinical suspicion and treatment.

## Acknowledgments

The authors thank the participating family and patient for allowing the case to be published.

## Author contributions

DV, a physician, participated in clinical management, conceptualized the study, drafted the initial manuscript, reviewed and revised the manuscript.

MLS was the attending physician for the case and she reviewed and revised the manuscript.

MJS participated in clinical management and reviewed and revised the manuscript.

BAT did the imaging studies, and reviewed the manuscript.

CP did the WES studies and reviewed the manuscript.

MLC, the principal investigator, coordinated the study, revised the manuscript, and conducted critical reviews of the manuscript for key intellectual content.

All authors contributed to drafting of the manuscript, approved the final version of the manuscript, and agree to be accountable for all aspects of the work.

**Conceptualization:** Daniel Almeida do Valle.

**Data curation:** Daniel Almeida do Valle, Mara Lúcia S. Ferreira Santos, Mônica J. Spinosa, Bruno A. Telles, Carolina Prando, Mara L. Cordeiro.

**Formal analysis:** Daniel Almeida do Valle, Mônica J. Spinosa, Bruno A. Telles.

**Investigation:** Daniel Almeida do Valle, Mara Lucia S. Ferreira Santos and Carolina Prando.

**Methodology:** Daniel Almeida do Valle, Mara L. Cordeiro.

**Supervision:** Mara L. Cordeiro.

**Writing – original draft:** Daniel Almeida do Valle, Mara Lúcia S. Ferreira Santos, Carolina Prando, Mara L. Cordeiro.

**Writing – review & editing:** Daniel Almeida do Valle, Mara Lúcia S. Ferreira Santos, Mônica J. Spinosa, Bruno A. Telles, Carolina Prando, Mara L. Cordeiro.
